# Primary healthcare and family medicine in Kenya

**DOI:** 10.4102/phcfm.v6i1.726

**Published:** 2014-12-01

**Authors:** Patrick Chege

**Affiliations:** 1 Department of Family Medicine, Moi University, Kenya

The Kenya Government's Health Sector Strategic Plan for 2005 to 2010, whose theme was ‘Reversing the Trends’, introduced six health service delivery levels and gave prominence to community-based services at level 1. Community-based primary healthcare services are aimed at empowering Kenyan households and communities to take charge of improving their own health.^1^ This is achieved by establishing a community care unit to serve about 5000 people and instituting well-trained Community-Owned Resource Persons (CORPs) who each serve 20 households. Every 25 CORPs work under a Community Extension Worker (CHEW). The recruitment and management of CORPs is carried out by village and facility health committees. This strategy was guided by extensive consultation amongst stakeholders who believed it was the best solution with regard to addressing health service inequities and would reverse the deteriorating health indicators. The objectives included: providing community services for all socioeconomic groups, building capacity of care givers and strengthening health facility–community linkages. This was rolled out in pilot districts, but is yet to cover all the Kenyan districts and/or counties. An evaluation of the programme in 2010 reported some degree of success, despite challenges.^2^ It is reported that many health workers in primary care facilities have not fully accepted these community-based health workers and have low levels of respect and collaboration.

In 2005, Moi University admitted the first three registrars for Masters in Medicine (MMed) in Family Medicine. By 31 December 2013, 20 had graduated from the programme. Of these, 15 are employees of the Ministry of Health and serving in rural district hospitals in Kenya, two are employed as lecturers by Moi University (both are also involved in clinical work in rural district hospitals) and the other three are working in rural Kenyan mission hospitals. Although based at district hospitals, the Family Physicians are collaborating with the county and/or district health managers to also focus on primary healthcare. However, the huge number of patients who need emergency and acute care at the district hospital level have made it difficult for the few family physicians to have a real impact at the primary care level.

Moi and the Aga Khan Universities are the only ones currently offering Family Medicine training in Kenya. The Aga Khan University is private and trains registrars, predominantly in Nairobi, within a referral hospital setting. It is hoped that public–private partnerships in the future will enable these registrars to also be trained in the district health services in what is referred to as ‘rural Family Medicine practice’ after two years on the Nairobi campus. Their initial group of registrars (admitted in July 2012) has yet to progress to that stage. Other universities that are at different stages of developing Family Medicine programmes include Maseno University, Kenyatta University and the Kabarak University. Kenyatta and Maseno are public universities, whilst Kabarak is a private one.

In 2007, stakeholders in the training of Family Medicine wrote a Family Medicine Policy document that was adopted by the Ministry of Health.^3^ The policy document defines the Family Physician and the envisaged role of this health worker in the public health system. The policy also establishes a Family Medicine Coordinating Committee chaired by the Director General of Health Services. It is hoped that this policy, together with an increased number of training programmes, will help family physicians to make a larger contribution to the health system in Kenya in the future.
